# Practical Hints

**Published:** 1862-07

**Authors:** J. D. White


Practical Hints.—*By J. D. White.*—Caries of the teeth has given rise to a great deal of speculation as well as experiment, and still young and old patients are losing their teeth, it seems, more rapidly than ever, spite of all that is known. Some authors have labored to divide caries into several different kinds, but there is but one kind—the only difference is in the degree of intensity with which it makes its progress in different individuals, and at different periods of life, varying with the habits and constitution of the patient. The variability of decay in some patients is so uncertain that it is impossible for a dentist to predict what will be the result for any considerable time ahead. It is very unwise for a dentist to tell his patients that in five or in ten years they will not have a tooth in their heads. The best thing a dentist can do is to impress upon the minds of his patients to keep constantly on the watch. We have a large number of patients with whom we make another or future appointment, when we have put their teeth in complete order—say from one month to six weeks or two months off. If such course were not pursued, they would either get the nerves exposed, or lose their teeth. We placed the teeth of a lady patient, as we thought, in good condition, on the eleventh of May, and gave her a clearance for two months until she would get over her usual attack of hay fever; but she called on us on Friday, the thirteenth of June, without an appointment, and there were three cavities discovered. This may seem strange, but the facts in the case may perhaps give some light on the subject. A few days after she returned home to her country place, she was attacked with a succession of white, ulcerated spots all over the mouth, which kept her in the most distressed condition imaginable. When these passed off, she thought it best to get her teeth examined, and the result was as stated above. It is not unfrequent that patients get out of confidence with their dentists. Because in a short time after having visited them they discover a decay, or some tooth becomes uncomfortable, they begin to question the carefulness of their former dentists, and seek the advice of others. Now here is a very difficult point to manage, and doubtless every dentist who has practiced a quarter of a century will agree to it.

A few weeks since, a young lady from a distant city, twenty-five years of age, called to get her teeth examined, when *ten* cavities were discovered. This *killed* her dentist for all time to come, in her mind. "He is getting too careless! He is too fond of going to parties. He is attending too much to pleasure, and that is the way I am losing my teeth!" and many other similar expressions fell fast from her lips. Now the truth is, this poor girl's teeth are never comfortable on account of the acidity of the mouth; and if her dentist had told her, or impressed it on her mind to pay him monthly visits, he would have saved himself the charges and have done his patient great service. He had plugged one cavity for the patient only three weeks before she made her call upon us. If the dentist had not been one for whom we entertain a high respect, and to whom we have referred patients for many years, the fact would not be of much value. We have just finished a series of operations of seventeen plugs for a young lady, nineteen years of age, from the same city. Now it seems that a dentist does not do justice to his patient or himself, when he places his teeth in good order and sends him away to return at his own pleasure.—*Dental Cosmos.*

